# Novel mutation in the *SLC12A3* gene in a Sri Lankan family with Gitelman syndrome & coexistent diabetes: a case report

**DOI:** 10.1186/s12882-017-0563-0

**Published:** 2017-04-26

**Authors:** Chandrika Jayakanthi Subasinghe, Nirmala Dushyanthi Sirisena, Chula Herath, Knut Erik Berge, Trond Paul Leren, Uditha Bulugahapitiya, Vajira Harshadeva Weerabaddana Dissanayake

**Affiliations:** 1 Endocrinology Unit, Colombo South Teaching Hospital, Kalubowila, Sri Lanka; 2 Human Genetics Unit, Faculty of Medicine, University of Colombo, Kynsey Road, Colombo 8, Sri Lanka; 3 Nephrology Unit, Sri Jayewardenepura General Hospital, Thalapathpitiya, Nugegoda Sri Lanka; 4 Unit for Cardiac and Cardiovascular Genetics, Department for Medical Genetics, Oslo University Hospital, Ullevaal, Oslo, Norway

**Keywords:** Genetics, Gitelman syndrome, Hypokalaemia, Salt-losing nephropathy, *SLC12A3*, Case report

## Abstract

**Background:**

Gitelman syndrome (GS) is a rare autosomal recessively inherited salt-wasting tubulopathy associated with mutations in the *SLC12A3* gene, which encodes for NaCl cotransporter (NCC) in the kidney.

**Case presentation:**

In this report, we describe two siblings from a Sri Lankan non-consanguineous family presenting with hypokalaemia associated with renal potassium wasting, hypomagnesemia, hypocalciuria and hypereninemic hyperaldosteronism with normal blood pressure. Genetic testing showed that both were homozygotes for a novel missense mutation in exon 10 of the *SLC12A3* gene [NM_000339.2, c.1276A > T; p.N426Y], which has not previously been reported in the literature in association with GS. Their mother was a heterozygous carrier for the same mutation. The father was not alive at the time of testing. This novel mutation extends the spectrum of known *SLC12A3* gene mutations and further supports the allelic heterogeneity of GS. Interestingly both siblings had young onset Diabetes with strong family history.

**Conclusion:**

These findings have implications in providing appropriate genetic counseling to the family with regard to the risk associated with inbreeding, the detection of carrier/presymptomatic relatives. It further expands the known spectrum of genotypic and phenotypic characteristics of Gitelman syndrome.

## Background

Gitelman syndrome [GS; OMIM #263800] is a rare inherited salt-losing tubulopathy characterized by hypokalaemic metabolic alkalosis, hypomagnesemia, hypocalciuria, and hypereninemic hyperaldosteronism with normal blood pressure. In 1966, Gitelman et al. [1] first described three adult female patients with Bartter syndrome (BS)-like symptoms accompanied by hypomagnesemia and hypocalciuria. The disease was found to be mostly associated with inactivating mutations in the *SLC12A3* gene located on chromosome 16q13, which encodes the thiazide-sensitive NaCl cotransporter (NCC), on the apical membrane of the distal convoluted tubules (DCTs) in the kidneys. It is recognized as a BS-like disease and commonly presents with features of hypokalaemia. BS and GS share common clinical and biochemical characteristics, but the urinary calcium to creatinine ratio is an index of urinary calcium excretion rate, which can be used to distinguish between the two syndromes [2]. Genetic heterogeneity exists and a minority of patients with GS phenotype harbour mutations in the *CLCNKB* gene which encodes the basolateral chloride channel type B in the distal nephron. *CLCNKB* gene mutations are typically seen in patients with classical BS phenotype [3, 4].

More than 140 mutations in the *SLC12A3* gene have been identified in patients with GS. The majority of the mutations are missense and nonsense mutations, but frameshift, splice-site, and intronic mutations have also been described [5]. GS is inherited as an autosomal recessive trait, and homozygous as well as compound heterozygous mutations have been identified [4–6]. It is reported that 18 to 40% of suspected GS patients carry only one *SLC12A3* mutant allele, with large genomic rearrangements accounting for the remaining unidentified mutations [5]. Most patients have onset of symptoms as adults, but some can present in childhood. Clinical features include transient periods of muscle weakness and paralysis, paresthesia, numbness, abdominal pains, polyuria, polydipsia and nephrocalcinosis [7]. Thiazide like action in GS associated hypokalaemia and hypomagnaesemia has been described to cause glucose intolerance and insulin secretion abnormalities [8] but data is limited on this association in large scale phenotype-genotype analysis studies except few case reports and a recently published small Chinese study [7–10].

GS is a rare inherited tubulopathy in the South Asian region and only few cases have been reported from Sri Lanka up to date. There is paucity of data on the genotypic and phenotypic characteristics of this disease in the Sri Lankan context. This paper describes the first reported non-consanguineous family with GS caused by a novel missense mutation in exon 10 of the *SLC12A3* gene with co-existent Diabetes.

## Case presentation

Two siblings (brother aged 31 years and sister aged 27 years) from a non-consanguineous family were clinically diagnosed to have GS. The male sibling first presented at the age of 16 years with acute onset hypokalaemic paralysis of lower limbs with a serum potassium level of 1.2 mmol/l (3.5-5.0 mmol/l). Further biochemical analysis showed associated urinary potassium wasting [Urinary K^+^ excretion (Spot) - 30 mEq/l], metabolic alkalosis (pH 7.56, HCO_3_^−^ - 30 mEq/l), hypomagnesemia [serum Mg^2+^ - 1.64 mg/dl (1.9-2.5 mg/dl)] and hypocalciuria [24 h urinary Ca^2+^ excretion – 88 mg/d (100-300 mg/d) and Ca/Cr ratio <0.07 (0.14-0.2)]. Despite having hyperreninemic [100 pg/ml (5.4-34.5 pg/ml)] hyperaldosteronism [368 pg/mL (49-176 pg//ml)], he was normotensive throughout. He was not on any medications such as diuretics or laxatives, which could lead to a similar clinical picture. His thyroid function was normal. His sister was otherwise healthy except for mild symptoms of occasional muscle weakness. On screening, at 18 years of age, she was found to have hypokalaemic metabolic alkalosis [serum K^+^ - 2.6 mmol/l (3.5-5.0 mmol/l), pH - 7.48, HCO_3_^−^ - 26 mEq/l] with hypomagnesemia [serum Mg^2+^ - 0.9 mg/dl (1.9-2.5 mg/dl) and hypocalciuria [24 h urinary Ca^2+^ excretion – 96.9 mg/d (100-300 mg/d) and Ca/Cr ratio 0.1 (0.14-0.2)]. She also had hyperreninemic [100 pg/mL (5.4-34.5 pg/ml)] hyperaldosteronism [213 pg/mL (49-176 pg/ml)], with normal blood pressure. In addition, both siblings were diagnosed to have Diabetes in the second decade of their lives. 

**Fig. 1:**
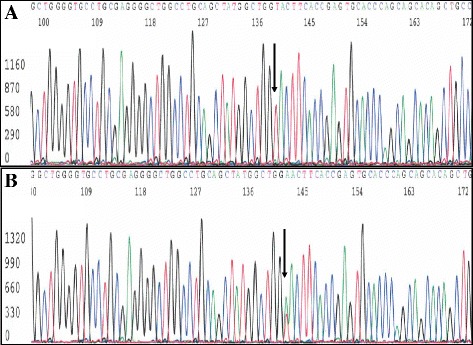
Partial electropherograms from exon 10 of the *SLC12A3* gene showing a homozygous missense mutation from A to T at nucleotide position 1276 [NM_000339.2, c.1276A > T; p.N426Y] in the proband (**a**) and the corresponding heterozygous mutation in the mother (**b**)

After providing pre-test counseling and obtaining written informed consent, both siblings underwent genetic testing for confirmation of the diagnosis of GS. Individual exons with flanking intron sequences of the *SCL12A3* gene were amplified from DNA extracted from EDTA-containing blood. The primers and conditions for thermal cycling are available upon request. The PCR products were purified using ExoSAP-IT (USB Corporation, Cleveland, OH) according to the manufacturer's instructions. Version 3.1 of the BigDye terminator cycle-sequencing kit (Applied Biosystems, Foster City, CA) was used for the sequencing reactions according to the manufacturer's instructions. The sequencing products were run on a Genetic Analyzer 3730 (Applied Biosystems) and analyzed using Secscape version 2.5 software (Applied Biosystems). Both siblings were found to be homozygotes for a novel missense mutation in exon 10 of the *SLC12A3* gene [NM_000339.2, c.1276A > T; p.N426Y] [Fig. 1]. Multiplex ligation-dependent probe amplification (MLPA) analysis of the *SLC12A3* gene (SALSA MLPA P136 Gitelman syndrome probemix) was also performed on these patients but did not show any large genomic rearrangements. Only their asymptomatic mother was alive, and after providing pre-test counseling and obtaining written informed consent, genetic testing showed that she was a heterozygous carrier for the same mutation. Their father was not alive at the time of testing, thus his carrier status could not be ascertained. There were no other clinically affected/unaffected family members who underwent genetic testing. However, given the fact that the mother was an unaffected carrier, it can be inferred that the father was also a carrier, and that the homozygous state is associated with the condition in the two children since both have the classic features of GS. This mutation has not previously been reported in association with GS, but another mutation in the same codon (p.N426K) has been found in a Chinese patient with GS phenotype [11]. This novel missense mutation was not found in the Exome Aggregation Consortium database (which comprises approximately 60,000 subjects), or in any of the other population specific databases (1000 Genomes, ESP). It was also absent in our existing database of de-identified Sri Lankan exome/genome sequences. The predicted effects of the mutation c.1276A > T in the *SLC12A3* gene results in a substitution of an asparagine (N) to tyrosine (Y) in residue 426 [Alamut Visual version 2.7 (Interactive Biosoftware, Rouen, France)], which gives rise to an amino acid change of large physicochemical difference. All in-silico tools predicted this variant as pathogenic with very high confidence scores (Mutation Taster; Polyphen2, SIFT, Provean). The mutation resides in a very highly conserved region of the *SLC12A3* gene (calculated and confirmed by two different algorithms: PhasCon and PhyloP). In addition, the asparagine in residue 426 is moderately conserved, when considering 13 species (Alamut). However, in the 10 species with same amino acid sequence surrounding residue 426, all but one have an asparagine in this position, indicating that this amino acid is of functional importance. The Asparagine to Tyrosine is a non-conservative amino acid substitution which may result in the alteration of the structure of the protein. Non-conservative amino acid substitutions are likely to be pathogenic. Even though functional studies were not carried out for characterizing the effects of the mutation on protein level, based on the bioinformatics data outlined above, it cannot be ruled out that homozygosity for the mutation can result in GS.

## Discussion

The phenotype of GS is highly variable in terms of age of onset, severity and nature of biochemical and clinical manifestations. This heterogeneity has even been described within families with identical genetic mutations [12]. Some GS patients are asymptomatic or present with mild weakness, while others show severe neuromuscular symptoms including hypokalaemic paralysis. Other reported uncommon symptoms include joint pains, constipation, polyuria, chondrocalcinosis and nephrocalcinosis. The main electrolyte abnormalities include hypokalaemia, metabolic alkalosis, hypomagnesemia and hypocalciuria. Patients experience these problems in variable severity and combinations. Many symptoms relate to these biochemical abnormalities, but not all cases with severe electrolyte abnormalities have clinical symptoms and vice versa [13]. These two siblings with an identical mutation had some phenotypic similarities, but they had significant heterogeneity in terms of disease severity. The male sibling was more severely affected with muscle weakness and paralytic episodes and an earlier onset of disease compared to his sister. Early onset of severe disease with muscle paralysis among males was a recognized feature in previously published cases of GS [6, 11]. Severe symptomatic hypokalaemia is also reported among females [7], but the female sibling described in this report did not present with severe symptoms. Both siblings were offered post-test genetic counseling and managed symptomatically with potassium, Mg supplementation and aldosterone antagonist therapy (spironolactone). During follow up, their K was maintained above 3 mmol/L and none of them had hospital admissions with major muscle problems, severe hypokalaemia or other GS associated symptoms.

Both patients were found to have coexistent Diabetes. Female sibling had glucose intolerance initially and later progressed to have Diabetes at the age of 20 years, while her brother developed Diabetes at the age of 19 years. A strong positive family history of Diabetes was present in 3 consecutive generations on the maternal side of the family. They were both lean and the features of insulin resistance: acanthosis nigricans were not evident in them. Female sibling was evaluated for the type of Diabetes and found that she was negative for Glutamic acid Decarboxylase (GAD) antibodies, and Islet Cell antibodies (ICA). She had elevated C-peptide levels [6.4 ng/ml (0.81-3.1 ng/ml)]. Therefore, the possibilities of Type 2 Diabetes and Maturity Onset Diabetes of Young (MODY) were considered, but genetic testing for further differentiation was not performed due to lack of facilities at our setting. Type 1 Diabetes has been very rarely reported with coexistent GS. These case reports have described it as a coincidence, rather than an association [9, 10]. In a Chinese study, a higher incidence of abnormal glucose metabolism, insulin secretion and type 2 Diabetes had been identified, when 16 GS patients were compared with healthy individuals. They had speculated this to be mostly contributed by hypokalaemia and hypomagnesemia in GS [8]. The mechanisms, by which hypomagnesemia induces or worsens diabetes are not well understood, but it is thought that chronic hypomagnesemia may induce altered cellular glucose transport, reduced pancreatic insulin secretion, defective post receptor insulin signaling, and altered insulin-insulin receptor interactions [14]. In addition, it has been shown that complications of any type of Diabetes are more severe in the presence of chronic hypomagnesaemia of any cause [14]. Studies have shown that hypokalaemia leads to impaired secretion of biologically active insulin, and insulin insensitivity due to unknown reasons [15]. MODY, which is a monogenic type of Diabetes, is not reported to be associated with GS in literature. However, a considerable proportion of HNF 1β associated MODY patients display hypomagnesaemia, which sometimes mimics GS clinically [16]. In the case of Diabetes also, our male patient had more severe disease and developed anti-hyperglycaemic treatment failure requiring insulin 5 years after diagnosis, but the female patient still remains well controlled on two anti-hyperglycaemic medications. During the follow up period, approximately 10 years after the diagnosis of Diabetes, the brother developed microvascular complications, while the female sibling still remains without any diabetic complications, 7 years after diagnosis.

## Conclusion

Compared to Caucasian populations, GS is a rare inherited tubulopathy in the South Asian sub region and only few cases have been reported from Sri Lanka up to date. This novel mutation extends the spectrum of known *SLC12A3* gene mutations and further supports the allelic heterogeneity of this disease. Molecular genetic diagnosis of GS in this family is beneficial in providing appropriate genetic counselling with regard to the risk associated with inbreeding, the detection of carrier/presymptomatic relatives. This report provides further evidence to support the association of GS with Diabetes. There is paucity of data between the mutation profile and clinical course of the disease in the Sri Lankan context, including the severity of electrolyte imbalance at presentation and after treatment, amount of potassium and/or magnesium replacement, response to treatment, and subjective symptoms. Further studies on the phenotype-genotype analysis of GS in Sri Lanka would be useful for the early detection and management of this disease in the country and in the South Asian sub region.

## Abbreviations

GS: Gitelman syndrome
BS: Barter syndrome
NCC: NaCl cotransporter
DCT: Distal convoluted tubule
MODY: Maturity onset diabetes of young
